# Systemic Inflammatory Response during Laparotomy

**DOI:** 10.1155/2014/674303

**Published:** 2014-08-05

**Authors:** Ahmad Mahamid, Basel Jabarin, Gidon Almogy

**Affiliations:** ^1^Department of General Surgery, Hadassah-Hebrew University Medical Center, P.O. Box 12000, 91120 Jerusalem, Israel; ^2^Department of General Surgery, Hillel Yaffe Medical Center, P.O. Box 169, 38100 Hadera, Israel; ^3^Faculty of Medicine, Technion-Israel Institute of Technology, P.O. Box 9649, 31096 Haifa, Israel

## Abstract

*Background*. The aim of this study was to analyze the influence of laparotomy on the systemic inflammatory response in human patients suffering from secondary peritonitis. *Study Design*. A prospective study investigating the levels of white blood cells, C-reactive protein, platelets, interleukin-six, and tumor necrosis factor-alpha during laparotomy in five patients who suffered from secondary peritonitis. Six venous blood samples were collected perioperatively from each patient. The data were summarized by descriptive statistics and presented in a box plot. The hypothesis was that laparotomy increases the systemic inflammatory response, as has been described in animal models in previous studies. *Results*. The median age of the patients in this study was 84 years, the male to female ratio was 2 : 3, and the mortality rate was 80%. The most common cause of generalized peritonitis was ischemia of the colon. Analysis of the data showed no significant changes in the level of plasma inflammatory mediators during the surgical procedure, except for the platelet count which showed a significant decrease (*P* = 0.001). *Conclusions*. In contrast to experience with animal models, laparotomy in human patients with secondary peritonitis did not significantly increase the systemic inflammatory response. Furthermore, it contributed in significantly decreasing some of the systemic inflammatory mediators.

## 1. Introduction

Acute peritonitis may be classified as primary (spontaneous), where an infection has been raised de novo within the peritoneum, or secondary, where the inflammation involving the peritoneum is the result of an identifiable primary process. Secondary peritonitis is one of the most common indications for urgent abdominal surgery. Despite great advancements in diagnostic tools, surgical equipment, and technique, management of patients with severe secondary peritonitis remains a surgical challenge, with major morbidity and a mortality rate over 50% in most series [1].

The systemic inflammatory response represented by white blood cells count, C-reactive protein levels, platelets count, interleukin-six, and tumor necrosis factor-alpha level was found to be increased during surgical management of peritonitis in an animal model, and when comparing the laparotomy to the laparoscopy approach, the inflammatory response was significantly higher in the first [2].

To the best of our knowledge all the data in the English literature concerning systemic inflammatory response during abdominal surgery for secondary peritonitis are derived from animal models.

The objective of our study was to define the characteristics of the systemic inflammatory system during urgent laparotomy due to secondary peritonitis in humans.

The levels of white blood cells, C-reactive protein, platelets, interleukin-six, and tumor necrosis factor-alpha were used to assess the systemic inflammatory response.

## 2. Patients and Methods

### 2.1. Study Design

A prospective study included five patients with a clinical diagnosis of secondary peritonitis, between January 2008 and July 2009, from Hadassah Medical Center, Ein Kerem Campus, Jerusalem, Israel. The research was conducted after receiving approval of the Institutional Review Board (IRB).

### 2.2. Sample Collection and Analysis

Six venous blood samples were collected from each patient throughout the period before, during, and after surgery. They are indicated here as T0 to T5: one hour before the anesthesia (T0), immediately after the anesthetic induction (T1), immediately after the abdominal wall incision (T2), one hour after the abdominal incision (T3), immediately after the abdominal wall closure (T4), and 48 hours after the abdominal wall closure (T5) (Table 1).

Each blood sample was tested for inflammatory mediators including white blood cells (WBC), C-reactive protein (CRP), platelets (PLT), interleukin-six (IL-6), and tumor necrosis factor-alpha (TNF-*α*).

WBC and PLT counting was done using a differential blood smear. CRP amount was determined using Latex tests. The plasma was separated from the blood samples using centrifugation at 3000 g at 4°C for 10 minutes immediately after withdrawal and stored at −20°C. TNF-*α* and IL-6 serum levels were determined by using commercially available IMMULITE 1000 Immunoassay System (Siemens, Siemens Medical Solutions Diagnostics, USA).

### 2.3. Statistics

The data were summarized by descriptive statistics and presented in box plots. Differences between measurements at two time points were analyzed by Wilcoxon Signed Rank Test and differences between several related samples were tested using the Friedman Test, if appropriate. *P* < 0.05 was considered statistically significant.

## 3. Results

Five patients were included in our study and their clinical details are summarized in Table 2. Median age of the patients in this study was 84 years (range: 22–93), male to female ratio was 2 : 3, and mortality rate was 80%.

The most common cause of generalized peritonitis was ischemia of the colon (three patients: in two cases it was induced by primary vascular insufficiency, while in the third case distal colon obstruction with extreme proximal bowel dilatation resulted in full thickness ischemia and necrosis). The fourth patient had small bowel ischemia and necrosis due to strangulation within an internal hernia, and the fifth patient had both small and large bowel perforation due to a motorcycle accident.

### 3.1. Plasma Levels Cytokines

**Figure 1:**
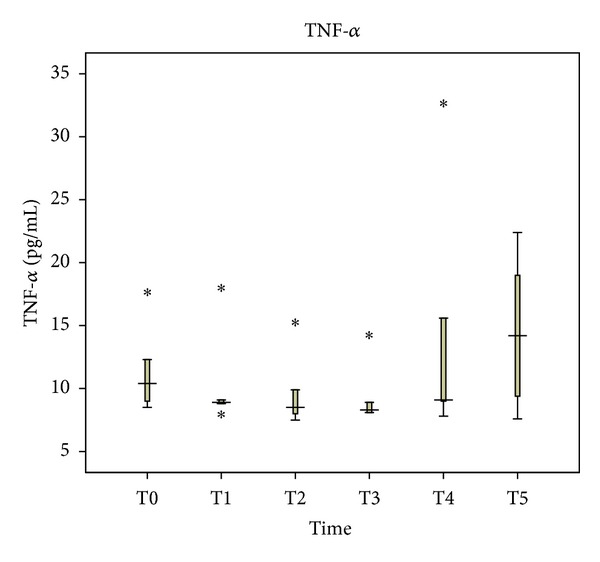
Perioperative tumor necrosis factor-alpha (TNF-*α*) levels in blood plasma.

**Figure 2:**
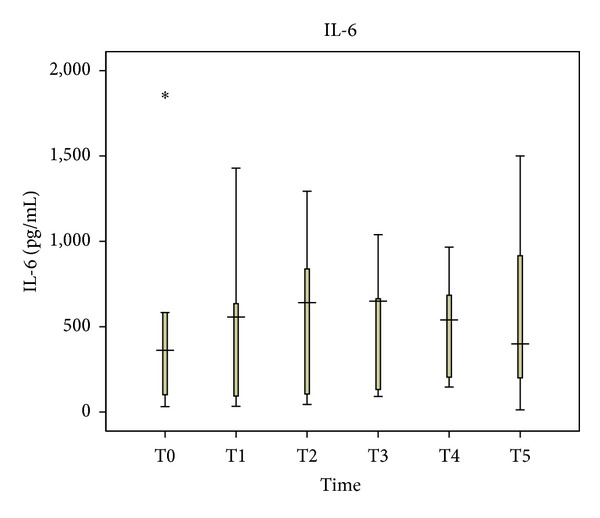
Perioperative interleukin-six (IL-6) levels in blood plasma.

In comparison with its levels one hour before anesthesia, plasma levels of TNF-*α* decreased insignificantly during surgery until immediately after the abdominal wall closure. Median values were 10.4 and 9.1 pg/mL (*P* = 0.273), respectively. At 48 hours after abdominal wall closure, a mild increase in TNF-*α* levels was noticed, with a median value of 14.2 pg/mL (*P* = 0.225) (Figure 1). In contrast, IL-6 increased during surgery from 362 pg/mL to 540 pg/mL (*P* = 0.5), ending with 400 pg/mL 48 hours after abdominal wall closure (*P* = 0.686) (Figure 2). The patterns of values distribution concerning TNF-*α* and IL-6 were not statistically different (*P* = 0.449 and *P* = 0.375, resp.).

### 3.2. Leukocyte Population

**Figure 3:**
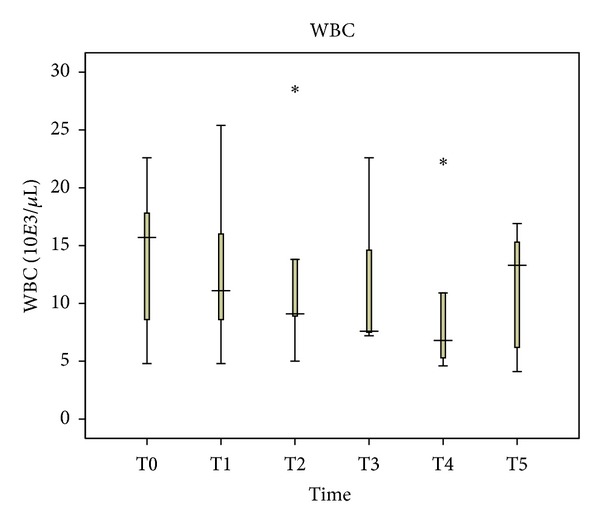
Perioperative white blood cells (WBC) levels in blood plasma.

The WBC counts decreased from 15.7 ∗ 10*E*3/*μ*L one hour before anesthesia, reaching 6.88 ∗ 10*E*3/*μ*L immediately after the abdominal wall closure (*P* = 0.138), and then increased to 13.3 ∗ 10*E*3/*μ*L at 48 hours after abdominal wall closure (*P* = 0.5). Despite the decrease of WBC counts during this period, its value was statistically insignificant (*P* = 0.483) (Figure 3).

### 3.3. Acute Phase Reactants

**Figure 4:**
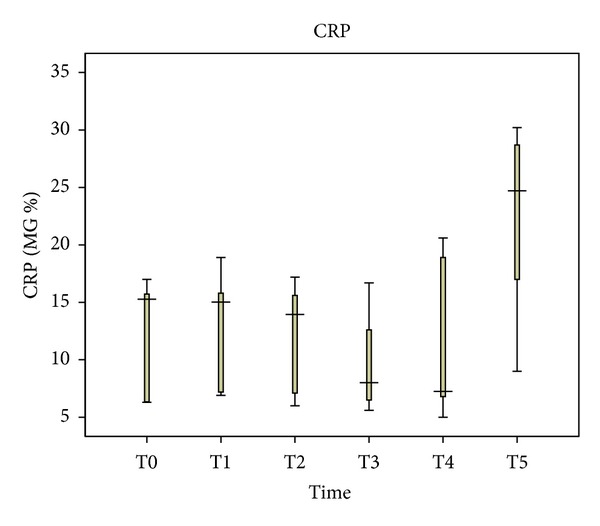
Perioperative C-reactive protein (CRP) levels in blood plasma.

CRP levels insignificantly decreased during surgery until immediately after abdominal wall closure. Median values were 15.27 and 7.25 mg%, respectively (*P* = 0.893). Then, the CRP levels started to increase, reaching 24.7 mg% at 48 hours after abdominal wall closure (*P* = 0.08). No statistically significant trend was shown in the CRP levels dynamics (*P* = 0.262) (Figure 4).

**Figure 5:**
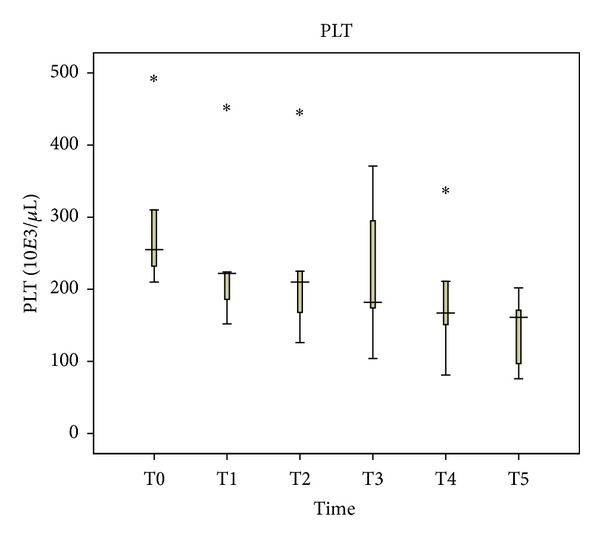
Perioperative platelets (PLT) levels in blood plasma.

PLT levels decreased significantly during the surgery (*P* = 0.001). They started as 255 ∗ 10*E*3/*μ*L one hour before anesthesia and reached 167 ∗ 10*E*3/*μ*L immediately after abdominal wall closure (*P* = 0.043). PLT counts continued to decrease to 161 ∗ 10*E*3/*μ*L 48 hours after abdominal wall closure (*P* = 0.043) (Figure 5).

## 4. Discussion 

Secondary peritonitis occurs most often after disruption of the integrity of the gastrointestinal tract. Despite great improvement in standards of diagnosis, antimicrobial therapy, and intensive care support, surgical treatment remains fundamental in the management of secondary peritonitis [3]. The operative approach is based on three basic principles: elimination of the source of the infection, reduction of peritoneal cavity bacterial contamination, and prevention of persistent or recurrent intraabdominal recolonization [4].

Despite optimal treatment, this life-threatening condition remains associated with high morbidity and mortality [5]. When generalized fecal peritonitis exists, the mortality rate varies and ranges from 50% to 100% [6]. Our mortality rate was 80%. The only survivor, out of the five patients, was a young healthy male, who had both small and large bowel perforation due to a motorcycle accident with mild peritonitis.

Peritonitis is defined as inflammation of the peritoneal cavity, where the peritoneal fluids increase in volume with the passage of a transudate rich in polymorph nuclear cells and fibrin [7]. Microbial contamination of the peritoneal cavity initiates the innate immune response. The balance between pro- and anti-inflammatory cytokines is thought to be related to the severity and outcome of peritonitis [8, 9].

The suggested mechanism of morbidity and mortality due to secondary peritonitis is a vicious circle, started by intraperitoneal inflammatory and toxic mediators. These induce vasodilatation and enhance the permeability of the visceral and parietal capillary vessels, thus facilitating the translocation of microorganisms, their toxic products, or cytokines from the peritoneal cavity to the circulation of the blood. This leads to a cascade of events: the onset is the systemic inflammatory response syndrome (SIRS), leading to the multiple organ failure syndrome (MOFS) and often ending with death.

Intraperitoneal cytokine measurements using an animal model of peritonitis have been suggested as early markers for adverse outcomes in patients with secondary peritonitis [10]. Surgical procedures in animal models with secondary peritonitis increased the systemic inflammatory response, especially when laparotomy was performed [2].

The theoretical explanation for this situation is that while opening the abdominal layers and thereby damaging the continuity of the histological tissue, we create a port of entry for the inflammatory mediators and microorganisms from the peritoneal cavity to the systemic circulation of the blood. This accelerates the vicious circle described above.

Analysis of our data showed no significant changes in the levels of plasma inflammatory mediators during surgical laparotomy, except for platelet count. Despite the decreasing trend of WBC, CRP, and TNF-*α* levels, they were statistically insignificant (*P* = 0.438, *P* = 0.262, and *P* = 0.449, resp.). On the other hand, the IL-6 levels showed an increasing trend during surgery but still without statistical significance (*P* = 0.375). The only significant change in the systemic inflammatory response identified in our study was attributed to the PLT count, which decreased during surgery (*P* = 0.001).

According to this data and in contrast to experience with animal models, laparotomy in humans with secondary peritonitis did not significantly increase the systemic inflammatory response. Furthermore, it contributed to significantly decreasing some of the systemic inflammatory mediators.

Our study had some limitations which must be considered: a small sample size due to logistic difficulties, no adjustment for the degree of peritonitis (mild, moderate, and severe), and type of peritonitis (purulent and fecal).

In conclusion, the systemic inflammatory response did not significantly change during laparotomy in humans suffering from secondary peritonitis. In order to have more solid and statistically significant data, a large, multicenter, and adjusted study is needed.

## Figures and Tables

**Table 1:** Timing of blood samples.

T0: one hour before the anesthesia.	
T1: immediately after the anesthetic induction.	
T2: immediately after the abdominal wall incision.	
T3: one hour after the abdominal incision.	
T4: immediately after the abdominal wall closure.	
T5: 48 hours after abdominal wall closure.	

**Table 2:** Clinical features of the patients.

Patient	Age (year)	Gender	Peritonitis cause	Mortality
F.A	22	Male	Small and large bowel perforation	No
D.S	84	Female	Large bowel ischemia	Yes
F.M	71	Male	Large bowel ischemia	Yes
S.L	93	Female	Small bowel ischemia	Yes
R.A	92	Female	Large bowel ischemia and obstruction	Yes
